# Use of PCR Signal and Therapeutic Drug Monitoring in a Switch Cohort Study to Tenofovir/Emtricitabine/Rilpivirine: A W96 Follow-Up

**DOI:** 10.1371/journal.pone.0134430

**Published:** 2015-07-30

**Authors:** Charlotte Charpentier, Minh Patrick Lê, Véronique Joly, Benoit Visseaux, Sylvie Lariven, Bao Phung, Patrick Yéni, Yazdan Yazdanpanah, Diane Descamps, Gilles Peytavin, Roland Landman

**Affiliations:** 1 IAME, UMR 1137, Univ Paris Diderot, Sorbonne Paris Cité, F-75018, Paris, France; 2 IAME, UMR 1137, INSERM, F-75018, Paris, France; 3 AP-HP, Hôpital Bichat-Claude Bernard, Laboratoire de Virologie, F-75018, Paris, France; 4 AP-HP, Hôpital Bichat-Claude Bernard, Laboratoire de Pharmacologie, F-75018, Paris, France; 5 AP-HP, Hôpital Bichat-Claude Bernard, Service de Maladies Infectieuses et Tropicales, F-75018, Paris, France; University of Pittsburgh Center for Vaccine Research, UNITED STATES

## Abstract

**Objective:**

To assess, in a clinical cohort, the efficacy of switching treatment in virologically-suppressed patients to tenofovir/emtricitabine/rilpivirine as a single-tablet regimen (STR) using the PCR signal of the viral load (VL) assay and plasma drug determination (C24h).

**Patients and methods:**

An observational single-centre study enrolling patients with VL<50 copies/mL initiating rilpivirine-based STR. C24h and VL were performed until W48 and W96 of STR, respectively. PCRneg was defined as an undetected PCR signal. Medians (IQR) were presented.

**Results:**

116 patients were enrolled. At STR baseline, time since first antiretroviral therapy and time of virological suppression were 6 years (2–9) and 17 months (7–43), respectively. Before STR initiation, patients were receiving protease inhibitors and non-nucleoside reverse transcriptase inhibitors-based regimen in 44% and 47% of cases, respectively. Historical genotype showed virus resistant to one drug of the STR in 6 patients (5%). At W96, 17 (15%) discontinued STR due to adverse events. The proportion of patients maintaining VL <50 copies/mL on treatment was 98%, 99%, 100%, 100%, 100% and 100% at W12, W24, W36, W48, W72 and W96, respectively. Among them, 70%, 66%, 68%, 59%, 74%, 68% and 60% were PCRneg at baseline, W12, W24, W36, W48, W72 and W96, respectively. Median rilpivirine C24h was 91 ng/mL (57–141, n = 285), with 91% of rilpivirine C24h >50 ng/mL, the target effective concentration.

**Conclusions:**

In this clinical cohort of virologically-suppressed patients switching to a new STR, most subjects had adequate rilpivirine C24h and displayed a high level of virological suppression with no residual viremia until W96.

## Introduction

Antiretroviral therapy recently evolved with the license of co-formulations as single-tablet regimens (STR) including two nucleoside reverse transcriptase inhibitors (NRTI) and: (i) one non nucleoside reverse transcriptase inhibitor (NNRTI) (efavirenz or rilpivirine); or (ii) one integrase inhibitor. STR strongly simplifies combined antiretroviral-based therapy (ART) with the objectives to improve quality of life and to favour long-term adherence. The efficacy of switching to tenofovir disoproxil fumarate/emtricitabine/rilpivirine has been assessed in the conditions of randomized clinical trials and in a population of moderately pre-treated patients.

A randomized clinical trial (GS 264–0106, SPIRIT study) assessed the efficacy of switching from a first-line protease inhibitor (PI)-based regimen to tenofovir/emtricitabine/rilpivirine STR [1]. At W24, 93.7% of the patients maintained virological suppression (HIV-1 RNA <50 copies/mL), showing the non-inferiority of this strategy compared to remaining under the initial ART [1]. An open-label study (GS 264–0111) assessed the efficacy of switching from first-line regimen tenofovir/emtricitabine/efavirenz to tenofovir/emtricitabine/rilpivirine in order to reduce central nervous system adverse events [2]. At week 48, 46 (93.9%) patients remained virologically-suppressed and virological failure occurred in 4.1% of the patients with no emergence of resistance [2].

However, limited data are available on the efficacy of switching to tenofovir/emtricitabine/rilpivirine in a clinical cohort of patients virologically-suppressed with a longer and a more diverse therapeutic history. Furthermore, efficacy of this strategy has not been assessed in an open clinical cohort on the residual viremia (i.e. detection of PCR signal), as well as with concomitant determination of antiretroviral plasma concentrations.

The aim of this study was to assess, in virologically-suppressed patients switching their antiretroviral-based regimen to the tenofovir/emtricitabine/rilpivirine STR, the proportion of patients displaying adequate drug plasma concentrations and undetectable viremia (i.e. with no PCR signal).

## Patients and Methods

### Study population

This is an observational single-centre cohort study enrolling all successfully ART-treated patients, i.e. with a VL <50 copies/mL, switching to tenofovir/emtricitabine/rilpivirine STR between September 2012 and February 2013. All the patients enrolled in this study gave their written informed consent to have their medical chart recorded in the electronic medical record system Nadis (Fedialis Medica, Marly Le Roi, France, CNIL number: 1171457 May 24^th^ 2006), designed for the healthcare medical follow-up of HIV-infected patients, that also included their agreement to participate to retrospective studies. All data were analyzed anonymously.

### Virological analysis

Historical resistance genotypes or therapeutic history were available in all patients. Genotypic resistance tests of RT region were performed according to the complete sequencing procedures and primers sequences described at www.hivfrenchresistance.org. Sequences were submitted to genotypic interpretation according to the Agence Nationale de Recherches sur le SIDA et les hépatites virales (ANRS) resistance algorithm (www.hivfrenchresistance.org, version 24).

Virological analyses were performed at week (W)12, W24, W36, W48, W72 and W96. We excluded the time-point at W4 due to the low sample size obtained. Plasma HIV-1 RNA quantification was performed using COBAS AmpliPrep/COBAS TaqMan HIV-1 Test, v2.0 (Roche Molecular Systems, Branchburg, NJ) with a limit of quantification of 20 copies/mL. PCRneg was defined as an undetected PCR signal, corresponding to an absence of residual viremia. We defined PCRpos when a PCR signal was detected below the limit of quantification of 20 copies/mL.

### Pharmacological analysis

Pharmacological analyses were performed during the first year of follow-up, at W4, W12, W24, W36 and W48. Plasma drug concentrations sampled 24 hours-post dose (C24h), were measured using Ultra Performance Liquid Chromatography combined with tandem mass spectrometry (UPLC-MS/MS) (Waters Corporation Milford, MA, USA) [3]. Rilpivirine C24h were interpreted according to the effective cut-off of 50 ng/mL, which approximately correspond to the minimal rilpivirine C24h (80+/-27 ng/mL) obtained in ECHO and THRIVE studies [4]. For tenofovir and emtricitabine the usual means C24h (+/-SD) were 44+/-22 ng/mL and 90+/-70 ng/mL, respectively.

### Renal function assessment

We assessed estimated glomerular function rate (eGFR) using MDRD equation values.

### Statistical analysis

Continuous variables were described by medians (25^th^ to 75^th^ percentiles) and qualitative variables by frequency and percentage.

## Results

### Patients’ characteristics

A total of 116 virologically-suppressed patients (67% male) were enrolled; characteristics are depicted in Table 1.

**Table 1:** Baseline characteristics of the 116 patients.

Characteristic	Value
Male sex, n (%)	78 (67)
Ethnicity (%)	
Caucasian	36
African	43
South America	21
Age, median years (IQR)	43 (39–50)
Active hepatitis co-infection, n (%)	
HBV (HBs Ag positive)	14 (12)
HCV (HCV RNA positive)	1 (1)
Duration of prior ART, median years (IQR)	6 (2–9)
Number of previous ART lines, median (IQR)	2 (1–3)
Duration of HIV-1 RNA <50 copies/mL before switch, median months (IQR)	17 (7–43)
Baseline CD4 cell count, median cells/mm^3^ (IQR)	630 (468–774)
Nadir CD4 cell count, median cells/mm^3^ (IQR)	230 (140–323)
Zenith HIV-1 RNA level, median log_10_ copies/mL (IQR)	5.08 (4.71–5.48)
Previous ART regimen, n (%)	
2 NRTI+ 1 NNRTI	54 (47)
EFV	41
ETR	11
NVP	2
2 NRTI + 1 PI/r	51 (44)
DRV	24
ATV	15
LPV	12
2 NRTI + RAL	5 (4)
Other ART	6 (5)
LPV/r monotherapy	2
TDF + RAL + MVC	1
RAL+ DRV/r	1
TDF/FTC/EFV + LPV/r	1
ATV/r + ETR	1
HIV-1 plasma tropism (n = 40), n (%)	
R5	30 (75)
Dual/Mixed or X4	10 (25)
HIV-1 subtype (n = 87), n (%)	
B	37 (43)
CRF02_AG	30 (34)
Other non-B subtypes	20 (23)

ART: antiretroviral therapy, ATV: atazanavir, DRV: darunavir, EFV: efavirenz, ETR: etravirine, FTC: emtricitabine, IQR: interquartile range, LPV: lopinavir, MVC: maraviroc, NNRTI: non nucleoside reverse transcriptase inhibitors, NRTI: nucleoside reverse transcriptase inhibitors, NVP: nevirapine, PI: protease inhibitors, RAL: raltegravir, TDF: tenofovir, /r: boosted with ritonavir.

Median CD4 cell count at time of tenofovir/emtricitabine/rilpivirine STR initiation was 630/mm^3^ (IQR = 468–774). Median time since first ART and median time with VL <50 copies/mL before STR initiation were 6 years (IQR = 2–9) and 17 months (IQR = 7–43), respectively. The median number of previous ART lines was 2 (IQR = 1–3). Before STR initiation, 54 (47%) of the patients were receiving a NNRTI-based regimen, 51 (44%) a ritonavir-boosted PI-based regimen, 5 (4%) a raltegravir-based regimen and 6 (5%) other ART combination. Regarding NNRTI-based regimens, the distribution was as follows: tenofovir/emtricitabine/efavirenz (n = 39), tenofovir/emtricitabine/etravirine (n = 10), abacavir/lamivudine/efavirenz (n = 2), abacavir/lamivudine/nevirapine (n = 1), zidovudine/lamivudine/nevirapine (n = 1) and abacavir/lamivudine/etravirine (n = 1). Among patients previously receiving PI-based regimen, 24 (47%), 15 (29%) and 12 (24%) were receiving darunavir, atazanavir and lopinavir, respectively. Among patients previously receiving a PI-based regimen, the previous NRTI backbone was: tenofovir/emtricitabine, abacavir/lamivudine and zidovudine/lamivudine in 44 (86%), 5 (10%) and 2 (4%) of the cases, respectively. The main reason of switching to rilpivirine-based STR was simplification (n = 70, 60%), but patients also switched for treatment-related adverse events: neuropsychiatric side effects (n = 22, 19%), cardio-vascular co-morbidities (n = 11, 10%), dyslipidemia (n = 8, 7%), and others (n = 5, 4%).

### Historical genotypes

**Fig 1:**
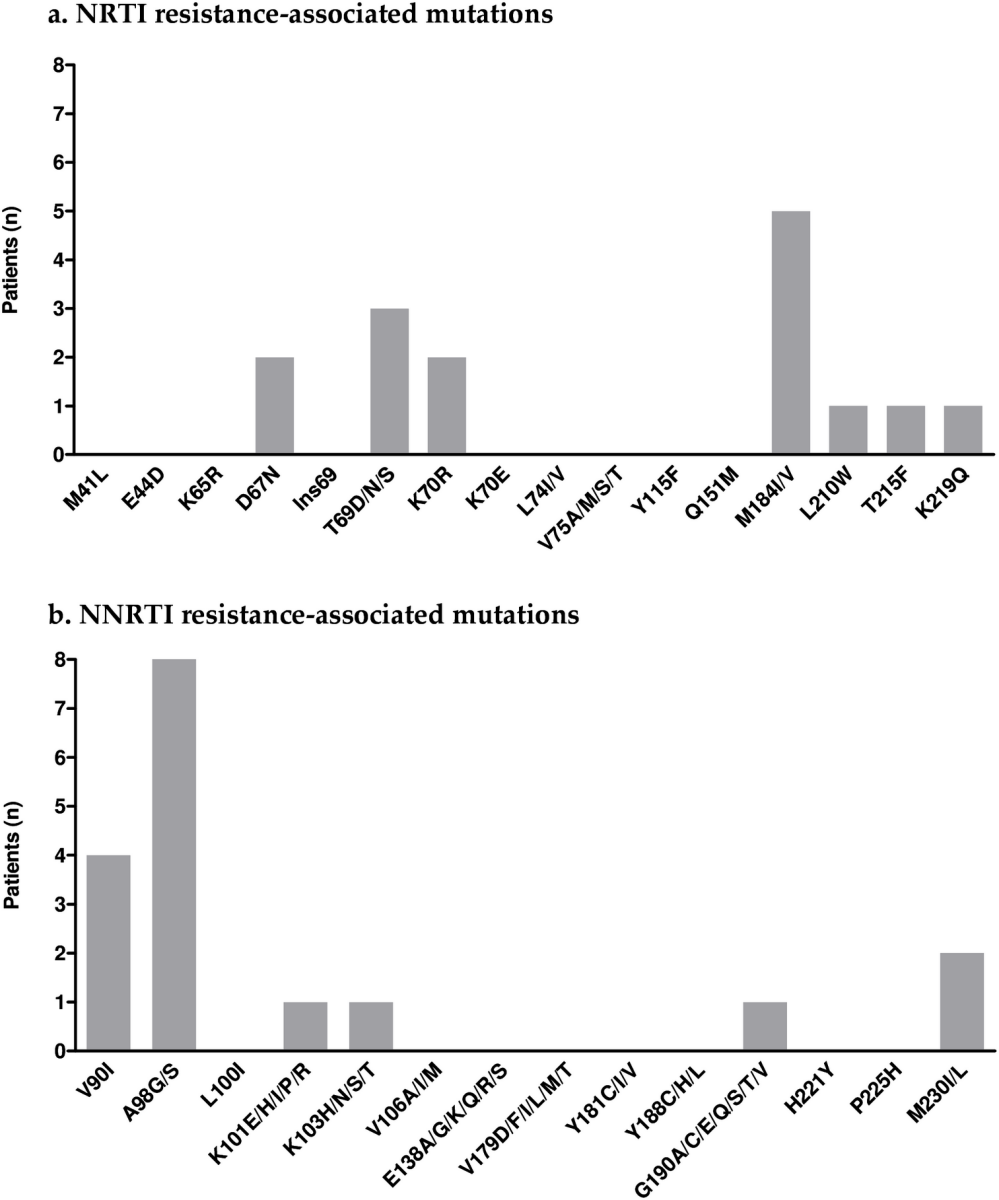
Distribution of NRTI (a) and NNRTI (b) resistance-associated mutations detected in the historical genotypic resistance tests of the patients. NNRTI: non nucleoside reverse transcriptase inhibitors, NRTI: nucleoside reverse transcriptase inhibitors.

Eighty-seven of the 116 (75%) virologically-suppressed patients had at least one plasma genotypic resistance test available during their therapeutic history, including 62 genotypes performed before first ART and 43 at time of a previous virological failure. Twenty-four patients (28%) displayed plasma virus with at least one NRTI or NNRTI resistance-associated mutation (RAM) (Fig 1). Among these latter, eight patients displayed viruses with NRTI RAMs: M184I/V (n = 5), T69D/N/S (n = 3), K70R (n = 2), T215D/F (n = 2), K219Q (n = 2), D67N (n = 1), L210W (n = 1) and 20 with NNRTI RAMs: A98G/S (n = 8), V90I (n = 4), V179I (n = 3), K101R (n = 2), K103N (n = 1), G190A (n = 1), M230I (n = 2). Three patients displayed virus with both NRTI and NNRTI RAMs. In six patients (5%) the presence of historical RAMs resulted in a genotypic resistance to at least one drug of the STR: three only to emtricitabine (M184I/V), one only to rilpivirine (M230I), one both to emtricitabine (M184V) and tenofovir (D67N-T69D-L210W-T215F) and one both to emtricitabine (M184V) and rilpivirine (M230I).

### Immuno-virological and pharmacological outcomes

In this observational study, plasma specimens were available in 107 patients at W12, 86 at W24, 66 at W36, 81 at W48, 88 at W72 and 77 at W96.

During the follow-up, 17 patients (15%) discontinued STR due to adverse events: 8 for neuropsychiatric side effects occurring between W4 and W72, 6 for renal toxicity between W48 and W72, 1 for drug-drug interactions with proton pump inhibitors, 1 for pregnancy and one for hyperlactatemia at W24 [5]. In addition, one patient decided himself to discontinue the STR at W60. Among the 8 patients with neuropsychiatric side effects, three had previously neuropsychiatric side effects with TDF/FTC/EFV and three different ones had rilpivirine C24h overexposure (107, 174 and 279 ng/mL).

**Fig 2:**
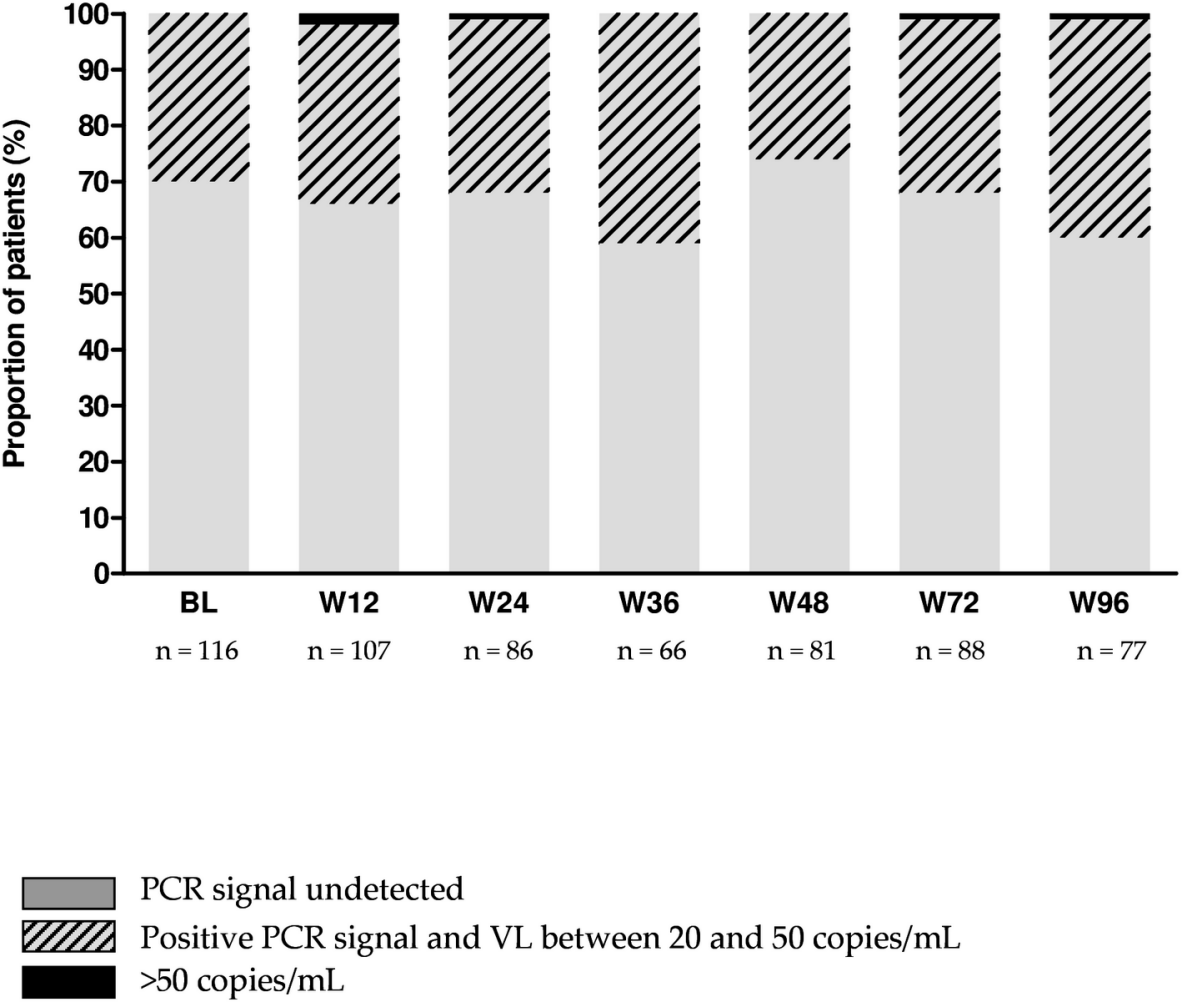
Distribution of plasma HIV-1 viral load at baseline (BL), Week (W)12, W24, W36, W48, W72 and W96.

**Fig 3:**
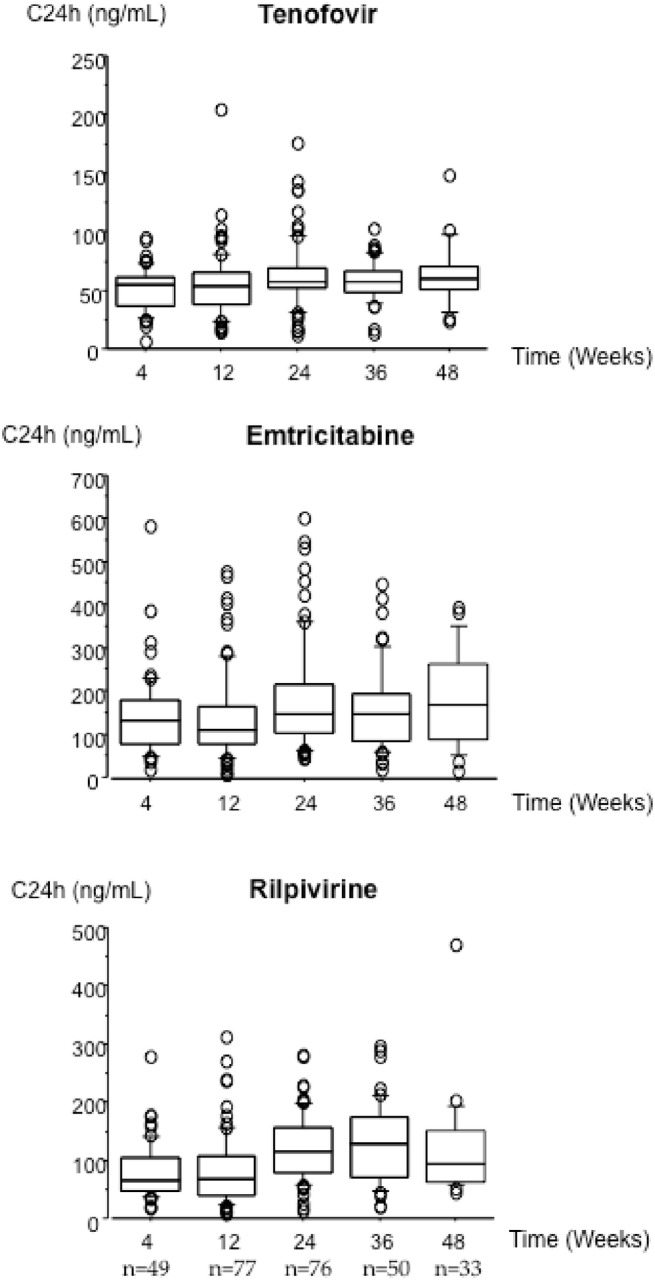
Distribution of tenofovir, emtricitabine and rilpivirine plasma concentrations at different time-points.

The number of patients still receiving tenofovir/emtricitabine/rilpivirine and maintaining plasma VL <50 copies/mL was 105 (98%), 85 (99%), 66 (100%), 81 (100%), 88 (100%) and 77 (100%) at W12, W24, W36, W48, W72 and W96, respectively (Fig 2). A total of 285 drug plasma concentrations were measured during the first year of STR. Overall, median tenofovir, emtricitabine and rilpivirine C24h were 56 ng/mL (IQR = 43–67), 132 ng/mL (IQR = 86–188) and 91 ng/mL (IQR = 57–141), respectively. During the first year of STR, 92%, 93% and 91% of tenofovir, emtricitabine and rilpivirine C24h were adequate or above the respective cut-offs (Fig 3). Inter-patient variability of tenofovir, emtricitabine and rilpivirine C24h was 44%, 69%, and 63%, respectively. Intra-patient variability of tenofovir, emtricitabine and rilpivirine C24h was 22%, 40% and 44%, respectively.

Regarding the six patients displaying resistant viruses to tenofovir, emtricitabine or rilpivirine in their historical genotypes, four were still receiving the STR at W96 (one had discontinued the STR and one with missing data) and they all maintained VL <50 copies/mL until W96 and all had adequate C24h during the 96 weeks of follow-up, except one patient at only one time-point.

The three patients having VL >50 copies/mL at W12 or W24 displayed VL values of 60, 70 and 75 copies/mL. No genotypic results could be obtained due to amplification failure. All of them had adequate C24h at all time-points of the study. At further VL determination, all three patients had VL <50 copies/mL.

Median CD4 cell count at W12, W24, W48 and W96 was 609/mm^3^ (IQR = 441–784), 635/mm^3^ (IQR = 465–793), 640/mm^3^ (IQR = 500–820) and 601/mm^3^ (483–738), respectively.

### Assessment of plasma residual viremia

The proportion of patients with no PCR signal (PCRneg) was 70%, 66%, 68%, 59%, 74%, 67% and 60% at baseline, W12, W24, W36, W48, W72 and W96, respectively (Fig 2).

Among the 39 patients with PCRneg at baseline and for whom VL data with PCR signal information were available at both W48 and W96, VL remained still PCRneg at W48 and at W96 in 82% (n = 32) and in 72% (n = 28) of the patients, respectively.

Regarding the six patients displaying resistant viruses to tenofovir, emtricitabine or rilpivirine in their historical genotypes, all had PCRneg at W48 of the STR. At W96, one patient discontinued the STR, one had missing data and all four remaining patients had VL <20 copies/mL including 2 PCRneg.

Regarding all paired VL and C24h data available at W24 (n = 73), the proportion of patients with adequate rilpivirine C24h, as >50 ng/mL, was 95% (n = 69). Among the four latter patients with suboptimal rilpivirine C24h, three had PCRneg, and one had a PCRpos. In addition, only one of them had also low tenofovir and emtricitabine C24h.

### Renal function follow-up

The median estimated glomerular function rate (eGFR) using MDRD equation values were stable over the study period: 94 mL/min/1.73m^2^ (82–112), 93 mL/min/1.73m^2^ (79–104), 93 mL/min/1.73m^2^ (82–103) and 90 mL/min/1.73m^2^ (80–102) at baseline, W24, W48 and W96, respectively. None of the patients displayed an eGFR value below 50 mL/min/1.73m^2^, whatever the time point. The mean decrease of eGFR between baseline and W24 was -4 mL/min/1.73m^2^ in the 61 patients with paired data at baseline and W24.

Regarding the 6 patients discontinuing STR for renal toxicity, they all showed a decreased eGFR with an increased blood creatinine level, added to proteinuria in 2 of them. The five patients with available C24h all showed tenofovir plasma overexposure (130, 156, 226, 319 and 564 ng/mL)

## Discussion

This is the first study assessing the efficacy on the residual viremia of a switch to tenofovir/emtricitabine/rilpivirine STR in virologically-suppressed patients (VL <50 copies/mL) issued from a clinical cohort with a follow-up of two years. Among the 116 patients receiving this new STR, most of them had adequate antiretroviral plasma concentrations and had a high level of virological suppression at W96 with no residual viremia.

In our clinical cohort 43% of the patients were African and 57% were infected with a “non-B” subtype. Half of them switched from a PI-based regimen and half from a NNRTI-based regimen. In our study an historical genotypic resistance test was available in 75% of the patients and was performed using population sequencing; thus we can hypothesize that we underestimated the prevalence of resistance in patients initiating tenofovir/emtricitabine/rilpivirine STR. In our study, patients displayed historical viruses with RAMs in 28% of cases but their presence resulted in a decreased susceptibility to one of the compound of the STR in 5% of the patients only. There was neither E138A nor K65R mutation, but the M184V mutation was detected in five cases in our population mainly receiving emtricitabine- or lamivudine-containing regimen. Most of the M184V mutation were evidenced in historical genotypes dating back for more than seven years and had a duration of virological suppression for more than six years The prevalence of the M184V mutation was similar to this observed in other observational cohorts assessing the switch to tenofovir/emtricitabine/rilpivirine STR in virologically-suppressed patients, ranging between 5 and 7% [6–9].

In our observational cohort, a high level of adherence associated with adequate antiretroviral plasma exposures with long half-lives was observed, and resulted in a high level of maintenance of the virological suppression with 100% at W48 and 100% at W96 of patients having VL <50 copies/mL. During the 2 years of follow-up, only three patients showed an isolated detectable VL >50 copies/mL. Thus, no virological failure or rilpivirine RAM emergence were observed in our study, contrary to that observed in the other observational cohorts [6–8]. These results were partly explained also by the systematic and large therapeutic education of patients before treatment initiating regarding the requirement of food and the avoidance of proton pump inhibitors due to rilpivirine-based STR. On a specific pharmacokinetic point of view, the very low within and between C24h variability would confirm the benefit of the favourable pharmacokinetic profiles of the three drugs in combination.

The high level of suppression observed in our study was similar to the findings of the randomized clinical trial SPIRIT assessing the switch to tenofovir/emtricitabine/rilpivirine STR compared to maintaining PI-based regimen in patients in success of first-line showing at W24 in snapshot analysis that 93.7% of patients maintained VL <50 copies/mL [1].

To our knowledge, virological follow-up using the PCR signal in patients switching to tenofovir/emtricitabine/rilpivirine has not been reported previously. In our study we showed that 70% of patients had no residual viremia at baseline before switching to the STR and that this proportion remained stable during the follow-up with 68% at W24, 74% at W48 and 60% at W96.

Seventeen patients (15%) of our cohort discontinued tenofovir/emtricitabine/rilpivirine STR for adverse events, a proportion similar to that observed in the other observational cohorts [6–8], but higher to that observed in the randomized trial SPIRIT in which 2.2% of the patients stopped tenofovir/emtricitabine/rilpivirine STR [1].

No modification in the renal function was observed in our study, however 94% of the patients were previously already receiving a tenofovir-based regimen before switching to tenofovir/emtricitabine/rilpivirine. Another observational cohort showed a more important change in the eGFR but the proportion of patients receiving tenofovir at time of STR initiation was lower in this study [7].

All drug-drug interactions (i.e. nonsteroidal anti-inflammatory drugs discontinued before initiating the STR regarding the presence of tenofovir) were advised at the start of our study.

In conclusion, our findings obtained in real-life settings confirmed data reported in the randomized trials. Indeed, in this clinical cohort assessing tenofovir/emtricitabine/rilpivirine STR switch in virologically-suppressed patients, we showed that all patients maintained VL<50 copies/mL at W96 and that more than 90% had adequate drug plasma concentrations. Finally, the proportion of patients with no residual viremia was stable around 60 to 70% during the two first years of tenofovir/emtricitabine/rilpivirine STR.

## References

[1] Palella FJ Jr, Fisher M, Tebas P, Gazzard B, Ruane P, Van Lunzen J, et al Simplification to rilpivirine/emtricitabine/tenofovir disoproxil fumarate from ritonavir-boosted protease inhibitor antiretroviral therapy in a randomized trial of HIV-1 RNA-suppressed participants. AIDS 2014;28:335–344. 10.1097/QAD.0000000000000087 24670520

[2] Mills AM, Cohen C, Dejesus E, Brinson C, Williams S, Yale KL, et al Efficacy and safety 48 weeks after switching from efavirenz to rilpivirine using emtricitabine/tenofovir disoproxil fumarate-based single-tablet regimens. HIV Clin Trials 2013;14:216–223. 10.1310/hct1405-216 24144898

[3] Jung BH, Rezk NL, Bridges AS, Corbett AH, Kashuba AD. Simultaneous determination of 17 antiretroviral drugs in human plasma for quantitative analysis with liquid chromatography-tandem mass spectrometry. Biomed Chromatogr. 2007;21:1095–1104. 17582235 10.1002/bmc.865

[4] Brochot A, De La Rosa G, Vis P, Corbett C, Vanveggel S, Cohen CJ, et al. Generalised additive modelling of virologic response to the NNRTIs rilpivirine (RPV, TMC278) and efavirenz (EFV) in treatment-naïve HIV-infected patients: pooled data from ECHO and THRIVE. In: Abstracts of the thirteen European AIDS Clinical Society AIDS Conference, Belgrad, Serbia, 2011. Abstract PS12/7.

[5] Nguyen LB, Harent S, Peytavin G, Visseaux B, Bastard JP, Dubourg O, et al Skeletal muscle toxicity associated with emtricitabine/rilpivirine/tenofovir fixed-dose combination: a case report. AIDS 2014;28:1995–1997. 10.1097/QAD.0000000000000387 25259710

[6] Gantner P, Reinhart S, Partisani M, Baldeyrou M, Batard ML, Bernard-Henry C, et al Switching to emtricitabine, tenofovir and rilpivirine as single tablet regimen in virologically suppressed HIV-1-infected patients: a cohort study. HIV Med. 2015;16:132–136. 10.1111/hiv.12183 25124291

[7] Reigadas S, Cazanave C, Bellecave P, Mazubert C, Le Marec F, Hessamfar M, et al One year follow-up of 275 virologically-suppressed patients switching to RPV/TDF/FTC, ANRS CO3 Aquitaine Cohort. Antivir Ther. 2014;19(Suppl 1): A122.

[8] Amiel C, Schneider V, Guessant S, Hamidi M, Kherallah K, Lebrette MG, et al Initiation of rilpivirine, tenofovir and emtricitabine (RPV/TDF/FTC) regimen in 363 patients with virological vigilance assessment in 'real life'. J Antimicrob Chemother 2014;69:3335–3339. 10.1093/jac/dku294 25114163

[9] Allavena C, Dailly E, Reliquet V, Bonnet B, Pineau S, André-Garnier E, et al Switching from tenofovir/emtricitabine and nevirapine to a tenofovir/emtricitabine/rilpivirine single-tablet regimen in virologically suppressed, HIV-1-infected subjects. J Antimicrob Chemother. 2014;69:2804–2808. 10.1093/jac/dku187 24907142

